# Expert opinion on detecting and treating depression in palliative care: A Delphi study

**DOI:** 10.1186/1472-684X-10-10

**Published:** 2011-05-27

**Authors:** Lauren Rayner, Annabel Price, Matthew Hotopf, Irene J Higginson

**Affiliations:** 1 Department of Palliative Care, Policy and Rehabilitation, Cicely Saunders Institute, King's College London, UK; 2 Department of Psychological Medicine, The Institute of Psychiatry, King's College London, UK

## Abstract

**Background:**

There is a dearth of data regarding the optimal method of detecting and treating depression in palliative care. This study applied the Delphi method to evaluate expert opinion on choice of screening tool, choice of antidepressant and choice of psychological therapy. The aim was to inform the development of best practice recommendations for the European Palliative Care Research Collaborative clinical practice guideline on managing depression in palliative care.

**Methods:**

18 members of an international, multi-professional expert group completed a structured questionnaire in two rounds, rating their agreement with proposed items on a scale from 0-10 and annotating with additional comments. The median and range were calculated to give a statistical average of the experts' ratings.

**Results:**

There was contention regarding the benefits of screening, with 'routine informal asking' (median 8.5 (0-10)) rated more highly than formal screening tools such as the Hospital Anxiety and Depression Scale (median 7.0 (1-10). Mirtazapine (median 9 (7-10) and citalopram (median 9 (5-10) were the considered the best choice of antidepressant and cognitive behavioural therapy (median 9.0 (3-10) the best choice of psychological therapy.

**Conclusions:**

The range of expert ratings was broad, indicating discordance in the views of experts. Direct comparative data from randomised controlled trials are needed to strengthen the evidence-base and achieve clarity on how best to detect and treat depression in this setting.

## Background

The management of depression in palliative care is complicated by the physical and psychosocial effects of advanced disease. The somatic symptoms of depression (e.g. fatigue, insomnia and poor appetite) mimic those of advanced illness, making it difficult to determine whether such symptoms are due to depression or physical disease [1]. Moreover, in palliative care it is difficult to differentiate depression from understandable fear and distress relating to declining health and impending death [2]. Antidepressant drugs and psychological therapies are the most common treatments for depression, yet there is limited evidence for their efficacy in palliative populations [3-5]. Further, studies suggest that in palliative care depression frequently goes untreated [6], and that interventions are often initiated so late that patients die before they have time to take effect [7].

The European Palliative Care Research Collaborative (EPCRC) recently produced a clinical guideline for the management of depression in palliative care to improve care for this population [8]. The purpose of the guideline is to provide recommendations on preventing, detecting and treating depression in palliative care, in order to aid decision-making, establish clinical policy and improve patient outcomes. Recommendations were devised using the best available evidence. Where evidence was equivocal or absent, expert opinion was sought. At the beginning of the project, an expert group was constituted to identify clinical priorities and offer knowledge, experience and opinion on contentious aspects of clinical practice. Literature scoping and initial discussions with the expert group revealed two main areas of uncertainty:

### 1) Choice of screening tool

Detection of depression in palliative care is bedevilled by a plethora of tools and a paucity of evidence [9]. Though few studies have shown screening to impact upon patient outcomes [10], it is advocated nonetheless as a simple, systematic and cost-effective way of improving identification of depression in medical settings [11]. To be useful to clinicians, screening tools must balance validity of assessment against brevity. Many tools were developed for use in physically healthy people and their applicability to palliative care is questionable [12]. The multitude of screening tools used in clinical practice and research attests to the current confusion surrounding the optimal method of detecting depression in palliative care [13].

### 2) Choice of treatment

Screening should always be complemented by effective treatment and a comprehensive management strategy [14,15]. Antidepressants have been shown to be efficacious and acceptable for treating depression in patients with life-threatening illness [16]. However, there is insufficient direct comparative data to recommend one antidepressant over others. Choosing an antidepressant for palliative care patients with multiple symptoms and medications is particularly challenging because of the increased risk of drug interactions and contraindications. Psychological therapy is the other recommended treatment for depression [11], but there are questions surrounding its feasibility and acceptability in palliative care. A recent Cochrane review found that psychotherapy was effective in treating depressive states in patients with advanced cancer [5]. However, there were not enough trials to draw conclusions regarding the relative efficacy of different types of therapy.

As there was insufficient evidence in the literature to guide choice of screening tool and choice of treatment, we conducted a Delphi study to evaluate expert opinion and inform the EPCRC guideline recommendations.

The following research questions were posed:

1) Which screening tool is most useful for detecting depression in palliative care?

2) Which antidepressant is most appropriate for treating depression in palliative care?

3) Which psychological therapy is most appropriate for treating depression in palliative care?

## Method

### Design

We used the Delphi method to assess expert opinion on screening for and treating depression in palliative care. Delphi is a formal consensus technique used to obtain and evaluate the views of an expert group with knowledge and experience in a specialised area. It has been found to be an effective method for determining the level of expert consensus on clinical questions and it is increasingly used in research [17]. Correspondence is carried out by email, making Delphi a feasible method for achieving consensus in a multinational expert group. Moreover, this allows anonymity and prevents prominent members of the group from exerting undue influence. Delphi is an iterative process involving repeated expert input and statistical description of responses. The aim is to elicit experts' views and encourage converging opinion on uncertain or contentious issues.

### Participants

An expert group was constituted to evaluate the perspectives and priorities of professionals with knowledge and experience in managing depression in palliative care. Expert nominations were sought via the European Association of Palliative Care (EAPC) board, the EAPC website and other palliative care associations in Europe. Experts were selected by the EPCRC guideline steering group based on their clinical expertise and research experience. To capture social, cultural and disciplinary differences, the Expert Group comprised 29 professionals from a range of disciplines and different countries (see Table 1).

**Table 1:** Background of the expert group

	*n *= 29
**Profession**	

Clinical psychologist	2
Psychiatrist	10
Physician	12
Psychotherapist	1
Nurse	1
Chaplain	1
Social worker	1
Pharmacist	1

**Present work setting**	

Clinical practice in palliative care	15
Research in palliative care	12
Psycho-oncology	1
Liaison psychiatry	1
CBT in physical illness	3
Social work	1
Oncology	4
Old age psychiatry	1
Pharmacy	1

**Gender**	

Female	13
Male	16

**Country**	

Canada	1
US	2
UK	16
Austria	1
Italy	3
Norway	1
The Netherlands	1
Spain	1
France	2
Germany	1

### Procedure

Items relating to choice of screening tool, choice of antidepressant and choice of psychological therapy were included in a structured questionnaire (see Additional file 1). These topics were chosen in consultation with the Expert Group. A list of key clinical questions was drafted by the EPCRC guideline development group (LR, MH, AP, IHJJ) based on the existing literature and emailed to the Expert Group who commented on its scope and relevance. The list was refined and experts were asked to rate the clinical importance of included items. The questions included in the Delphi questionnaire were those rated most highly by the expert group.

The Delphi method was applied in two rounds. In the first round, the questionnaire was emailed to the expert group with a request for members to rate their level of agreement with each item on a scale from 0 to 10. The experts were invited to leave comments to annotate their ratings and to suggest additional items to be included in the second round. In the second round the median and range of ratings were reported to the group together with anonymised comments. Items suggested by experts in the first round were added to the Delphi questionnaire, which the expert group was requested to complete again in light of the results of the first round. The median and range reported below are from the second round of Delphi as this represents the final view of the expert group.

## Results

Eighteen of the 29 experts (62%) returned a questionnaire in one or both rounds of the Delphi exercise. Respondents included seven palliative care physicians, six psychiatrists (all with experience working in palliative care), two psycho-oncologists, a behavioural psychotherapist, a clinical psychologist and a general practitioner.

### Screening

After the second round of Delphi, the Hospital Anxiety and Depression Scale (HADS) (median 7.0, range 2-9) [18], the single question "Are you depressed? (median 7.0, range (0-10) [19] and two questions assessing low mood and low interest (median 7.0, range 1-10) [20] were rated as the most useful screening tools for detecting depression in palliative care (see Table 2). However, the experts' comments indicate some concern about the specificity of such screening tools: "It does sort of work, but pretty non-discriminating"; "Not really specific". Screening for depression using a generic symptom assessment tool (such as the Palliative Outcome Scale (POS) [21-23]) was also considered a useful method of detection by the expert group (median 6.5, range 1-10): "...utilizes open questions within a conversation about many other symptoms, hence less fear, less defence in answer...". Interestingly, the item "Routine informal asking" (suggested by an expert in the first round and included in the second iteration of the questionnaire) was rated more highly than any of the formal screening tools in the second round of Delphi (median 8.5, range 0-10). The Brief Edinburgh Depression Scale (BEDS), the Prime-MD PHQ-9, the Beck Depression Inventory (BDI) and the Distress Thermometer each achieved a median rating of 5.0 or lower. The experts' comments indicate that the Prime-MD PHQ-9 and the Beck Depression Inventory (BDI) may be more suitable for assessing the severity of depression than for screening: "After screening, if practitioner is experienced and confident to use, but this is really for specialist psychiatric services"; "For assessment"; "It's a severity tool...".

**Table 2:** Experts' ratings: Method of detection

*Method* 0 = not useful, 10 = very useful	*Experts' comments*
Routine informal asking* Round 2: Median 8.5 (0-10); n = 10	-It is entirely appropriate to ask patients about their mood as part of a comprehensive symptom assessment

**Two-items (low mood & loss of interest)** Round 1: Median 8.0 (4-10); n = 13 Round 2: Median 7.0 (2-9); n = 13	-Too rigid, it seems the shortest summary of a long screening questionnaire -There is evidence to support this -We are talking about screening, not assessment: if patient presents with a symptom then full assessment, but if screening otherwise mentally well population then this is a useful tool

**Hospital Anxiety and Depression Scale** Round 1: Median 7.0 (1-10); n = 13 Round 2: Median 7.0 (1-10); n = 13	-Long to complete for palliative care patients -Have used it in palliative care patients very successfully -I don't find this difficult to use in palliative care in hospice or hospital setting, even for pretty weary patients -Is just a screener for emotional distress, not useful in diagnosing depression -Very useful, very acceptable, very under-rated. For some reason the palliative care community have got it in for the HADS, but I don't think there is any evidence that any other measure is better, and quite a few are a lot worse -Less clear distinction between depression and anxiety

**Single-item "Are you depressed?"** Round 1: Median 5.0 (0-10); n = 13 Round 2: Median 7.0 (0-10); n = 12	-I know the limitations, but in my experience helpful in daily practice -Not really specific -Too direct and one closed question is not enough screening -Chochinov's first paper was based on a false premise. It does sort of work, though, but pretty non-discriminating

**As part of generic symptom assessment (e.g. POS, ESAS)** Round 1: Median 7.0 (2-10); n = 13 Round 2: Median 6.5 (1-10); n = 14	-Good just as first wide screening, utilizes open questions within a conversation about many other symptoms, hence less fear ... less defence in answer, nevertheless not enough for a full screening -These aren't at all bad -Screening by definition has to be simple and quick. Don't confuse screening with severity assessment, there needs to be a 2 stage process: 1) screen; 2) if patient screens positive then use a tool to assess severity

**Brief Edinburgh Depression Scale (BEDS)** Round 1: Median 8.0 (2-10); n = 9 Round 2: Median 5.0 (1-9); n = 12	-Easy to use -The simplified version; but the 6 sentences are too crude for our patients

Beck's Depression Inventory* Round 2: Median 5.0 (0-8); n = 12	-Largely about thinking style and content and thus relatively un-skewed by physical problems. A better scale for measuring progress than HADS -Better use only for assessment -After screening, if practitioner is experienced and confident to use, but this is really for specialist psychiatric services

**Prime-MD PHQ-9** Round 1: Median 5.0 (0-10); n = 11 Round 2: Median 3.0 (0-9); n = 11	-Too complex -Too long -For assessment -It's a diagnostic tool, but it's pretty acceptable and not very difficult to use - It's a severity tool, it is easy to use, primary care practitioners will be familiar with this

**Distress Thermometer (DT)** Round 1: Median 6.0 (0-8); n = 9 Round 2: Median 2.0 (0-10); n = 13	-Distress and depression are different -Useful screen for generalists to decide whether to refer for specialist help -Useful to integrate depression in routine screening of other symptoms, mixed with distress, hence perceived by patients not as psychiatric inquiry.

*Items suggested by experts in the first round and rated by the group in the second round

### Antidepressant treatment

Mirtazapine (median 9.0, range 7-10) and citalopram (median 9.0, range 5-10) were considered the best choice of antidepressant for treating depression in palliative care (see Table 3). Venlafaxine (median 8.0, range 0-9), sertraline (median 7.5, range 0-10) and escitalopram (median 7.0, range 0-10) were also rated as useful by the expert group. Of all the drugs, the range of ratings was smallest for mirtazapine (7-10), indicating expert agreement regarding its advantages for this patient group. This was reflected in the additional comments about mirtazapine: "My first-line - evidence is that it is more effective than SSRIs and appetite stimulant and sedative qualities are useful side effects"; "...good profile regarding side effects, at this time most used".

**Table 3:** Experts' ratings: Choice of antidepressant

*Drug* 0 = not useful, 10 = very useful	*Experts comments*
**Mirtazapine** Round 1: Median 9.0 (7-10); n = 11 Round 2: Median 9.0 (7-10); n = 11	-Some data, good profile regarding side effects, at this time most used -Seems well tolerated -When side effects on sleep and appetite are handy -Too soft -Has useful sedative properties and well tolerated -My first-line - evidence is that it is more effective than SSRIs and appetite stimulant and sedative qualities are useful side effects

**Citalopram** Round 1: Median 9.0 (5-10); n = 11 Round 2: Median 9.0 (5-10); n = 13	-Side effects due to its antIHJJistaminic action

**Venlafaxine** Round 1: Median 7.5 (0-10); n = 8 Round 2: Median 8.0 (0-9); n = 13	-Less side effects than tricyclics -Useful if patient needs help for sedation -If depression is associated with chronic high level of anxiety -Not straightforward - discontinuation symptoms common. Cardiac toxicity

**Sertraline** Round 1: Median 7.0 (2-10); n = 11 Round 2: Median 7.5 (0-10); n = 12	-Favourable side effect profile -Interaction with dopamine system could give different and more side effects than other SSRIs -Least adverse effects, presently best available

**Escitalopram** Round 1: Median 8.0 (0-10); n = 10 Round 2: Median 7.0 (0-10); n = 13	-Not cost effective alternative to citalopram -Better tolerated than R-citalopram and less interaction with cytocrome 450 2D6

**Amitriptyline** Round 1: Median 5.0 (1-9); n = 10 Round 2: Median 4.0 (0-8); n = 12	-Side-effects -Too sedative, effect too delayed, frequent compliance obstacle due to side effects -If indicated for neuropathic pain -For individual TCAs I don't think there is sufficient evidence to differentially rate, although clinically I would favour those drugs that are used most often in palliative care anyway e.g. amitriptyline -An excellent antidepressant

**Paroxetine** Round 1: Median 5.5 (0-9); n = 10 Round 2: Median 3.0 (0-7); n = 13	-Too many pharmacological interactions -Interactions and withdrawal problems -No place - withdrawal syndrome

**Fluoxetine** Round 1: Median 3.0 (0-6); n = 10 Round 2: Median 3.0 (0-6); n = 13	-Too long half time, not useful for physically ill people -Long t1/2 and interactions -Too disinhibitory, but not better antidepressant than other SSRIs

**Imipramine** Round 1: Median 5.0 (0-6); n = 7 Round 2: Median 2.0 (0-8); n = 11	-Just in patients with depressive history who have already utilized before

**Nortriptyline** Round 1: Median 2.0 (0-9); n = 9 Round 2: Median 2.0 (0-7); n = 11	-If indicated for neuropathic pain -Less than amitriptyline

**Mianserin** Round 1: Median 5.0 (0-9); n = 7 Round 2: Median 0.0 (0-7); n = 11	-Little used in practice compared with other drugs and little evidence available -No point really - it is pharmacologically similar to mirtazapine, which doesn't hit the white cells

Though no TCA scored highly in the Delphi study, amitriptyline achieved the highest median score (median 5.0, range (1-9) probably on account of its efficacy in alleviating neuropathic pain:; "...clinically I would favour those drugs that are used most often in palliative care anyway e.g. amitriptyline"; "If indicated for neuropathic pain". The least favoured SSRIs were fluoxetine (median 3.0, range 0-6): "Too long half-life, not useful for physically ill people", and paroxetine (median 5.5, range 0-9): "Interactions and withdrawal problems". Of all the antidepressants, mianserin was rated most poorly: "No point really - it is pharmacologically similar to mirtazapine, which doesn't hit the white blood cells".

### Psychological therapy

Cognitive behavioural therapy was the experts' preferred choice of psychological therapy (median 9.0, range 3-10), followed by problem-solving therapy (median 8.0, range 3-10) (see Table 4). The item "Presence of a spiritual assistant upon request" was suggested by an expert in the first round and then achieved the third highest rating in the second round (median 7.5, range 1-10). Comments included: "We have to get better at specifically enquiring about the presence of a spiritual dimension to distress"; "Not for all patients - just on request". Mindfulness-based therapy, guided imagery and narrative therapy each achieved a median rating of 7.0.

**Table 4:** Experts' ratings: Choice of psychological therapy

Therapy 0 = not useful, 10 = very useful	Experts comments
**Cognitive Behavioural Therapy (CBT)** Round 1: Median 9.5 (4-10); n = 12 Round 2: Median 9.0 (3-10); n = 12	-Choice depends on therapist's expertise and patient's preference!! -Although psychotherapy at the end of life has some evidence base, there is less evidence for the effectiveness of individual therapies, so I feel unable to rate. -Is there any evidence anywhere that any of the below are effective interventions, and do no harm. Too much unproven 'counselling' has caused harm over decades that I would not want my patients and their carers exposed to this

**Problem-solving Therapy** Round 1: Median 10.0 (1-10); n = 11 Round 2: Median 8.0 (3-10); n = 12	-Probably better chance than CBT -Too superficial

Presence of spiritual assistant upon request* Round 2: Median 7.5 (1-10); n = 9	-We have to get better at specifically enquiring about the presence of a spiritual dimension to distress -Not for all patients - just on request

**Mindfulness-based therapy** Round 1: Median 7.0 (0-10); n = 9 Round 2: Median 7.0 (0-10); n = 10	-Useful for those who can learn it: interestingly, 'living in the moment' is a strategy spontaneously adopted by many patients who are coping well under difficult circumstances -Not sure if there's any evidence

**Guided imagery** Round 1: Median 6.0 (1-10); n = 11 Round 2: Median 7.0 (3-9); n = 9	-Temporarily relieves symptoms - but empowering and engenders hope -Sometimes impossible for cultural or age resistance

**Narrative Therapy** Round 1: Median 5.0 (0-8); n = 9 Round 2: Median 7.0 (0-9); n = 9	-I have experience: running well, easily accepted - especially by aged people - empowers the patient and increases his/her communication with loved ones -Not sure if there's any evidence

Music Therapy* Round 2: Median 6.0 (0-8); n = 9	-For those who cannot be reached by words -More evidence for relief of physical symptoms than depression

**Interpersonal Therapy** Round 1: Median 5.5 (0-10); n = 10 Round 2: Median 5.0 (0-8); n = 10	-Probably too time-consuming

**Couple therapy** Round 1: Median 5.0 (1-8); n = 12 Round 2: Median 4.0 (0-8); n = 11	-Can be very useful -Useful just if the family is reduced to two relevant people

*Items suggested by experts in the first round and rated by the group in the second round

Overall, fewer experts responded to the psychological therapy question, possibly on account of the expert group including fewer psychologists than psychiatrists. The lower response to this question may also indicate difficulty rating items in the absence of evidence: "Although psychotherapy at the end of life has some evidence base, there is less evidence for the effectiveness of individual therapies, so I feel unable to rate".

## Discussion

### Screening

A single question assessing depression [19] and two questions assessing low mood and loss of interest [20] were rated as moderately useful by the expert group (median 7.0). However, studies have shown that whilst these items are effective at excluding depression in the non-depressed, they are less successful at confirming cases [24], and this was reflected in the experts' comments. Nevertheless, some clinicians valued these 'ultra-short' screening tools: "I know the limitations, but in my experience helpful in daily practice". Of the longer screening tools, the HADS was rated most highly (median 7). Though one expert felt that the HADS was too long for palliative patients, a number commented that they had used it successfully in this setting.

Our initial objective was to determine the optimal screening tool for detecting depression in palliative care, yet perhaps the most important finding was that the experts rated 'informal routine asking' as more useful than administering a specified screening tool. Clinicians and patients may feel more relaxed and open if mood is considered in the context of a general conversation about symptoms, coping and well-being. This 'holistic' approach allows patients to tell their story and emphasise the issues important to them [25]. It is possible that a more rigid 'tick-box' approach may detract from deeper engagement with patients' problems.

Screening for depression has been recommended by several influential bodies [11,26]. However, research on screening has focused on tool development and validation [27,28], and the less easily evaluated yet fundamental question of whether screening improves patient outcomes remains unanswered [29]. We know of no randomised controlled trials assessing the impact of screening in palliative care, though there is nothing to suggest that its effects would be different from that found in other patient groups. A meta-analysis by Gilbody et al showed that introduction of depression screening in non-mental health settings was associated with a modest increase in the detection of depression (RR 1.27 (CI 1.02-1.59)), but had no effect on depression outcomes (Standardised mean difference -0.02 (CI -0.25-0.20)) [10]. However, this review only included studies that evaluated screening alone without further enhancement of care (e.g. case managers, nursing interventions, collaborative care). Identifying distress is just one link in a chain of actions needed to optimally manage depression. Improving patient outcomes requires a comprehensive system of care that addresses each link: detection, diagnosis, assessment, appropriate referral and effective intervention. Whilst screening for depression is useful as a catalyst to consider subsequent action, its intrinsic value is unclear. We assessed experts' preferred methods of detecting and treating depression in palliative care, but we did not address the decisions and actions required after detection to instigate appropriate and effective intervention. Future studies would benefit from a qualitative component exploring if and how positive screening outcomes influence treatment decisions and patient care. Further, data on the proportion of potential cases followed up with a diagnostic interview could elucidate the apparent gap between screening and subsequent benefit for the patient.

### Antidepressant treatment

Of the eleven antidepressants rated by the expert group, five achieved a median rating of 7 or higher, whilst six were rated 4 or lower. This shows a clear distinction between the antidepressants considered to be a good choice for treating depression in palliative care (mirtazapine, citalopram, venlafaxine, sertraline, escitalopram), and those considered less appropriate (amitriptyline, paroxetine, fluoxetine, imipramine, nortriptyline, mianserin). Whilst there was some consensus about choice of antidepressant, this should not be mistaken for clear evidence that one treatment is more effective than others. Such evidence can only be achieved from a randomised controlled trial, and evidence from the Delphi exercise is by comparison weak. However, with the exception of citalopram, the antidepressants rated highly by the expert group matched those found to be most efficacious in Cipriani's recent meta-analysis comparing 12 new-generation antidepressants. Of the four drugs identified as most efficacious in this meta-analysis, sertraline and escitalopram were better tolerated than mirtazapine and venlafaxine [30]. The extent to which Cipriani's findings from trials in physically well people can be extrapolated to patients receiving palliative care is open to debate. In palliative care, the coexistence and interaction of multiple symptoms and treatments complicates choice of antidepressant. The side effects of specific antidepressants may alleviate or exacerbate different symptoms of advanced disease. For example, tricyclic antidepressants can relieve neuropathic pain [31], but are generally contraindicated for patients with heart disease or liver failure [32]. The side effects of mirtazapine include sedation and weight gain [33], which are undesirable for most physically well people, but benefit many palliative care patients, particularly those with loss of appetite, a major concern for many patients and their families.

In addition to the present Delphi study, the authors have conducted a meta-analysis of antidepressants for depression in physical illness [4] and reported a more detailed analysis of those studies which examined depression in life-threatening illness [16]. Our review showed that antidepressants were more effective than placebo in treating depression in this context. There were not enough trials to enable us to assess the efficacy and acceptability of specific antidepressants, but we did conduct a subgroup analysis comparing classes of antidepressant. All the studies compared individual antidepressants with placebo and did not make direct comparisons between different antidepressants, therefore it is difficult to draw conclusions on the relative tolerability and effectiveness of classes of antidepressants, Nevertheless, indirect comparison suggested that in these populations there were no obvious differences between SSRIs and tricyclics in terms of drop out from treatment, and the TCAs were possibly more effective. The combined effect size for the three trials using mianserin or mirtazapine exceeded that observed for both SSRIs and TCAs. This gives some indirect credence to the expert group's high rating of mirtazapine, though comparative trials are needed before conclusions can be drawn.

### Psychological therapy

In line with current NICE guidance [11,34], the expert group rated CBT as the most useful psychological therapy for depression in palliative care. Though there is good evidence for CBT in people without physical illness, there is a paucity of data in palliative care [35,36]. A systematic review by Coventry et al identified two RCTs showing CBT to reduce depressive symptoms in COPD patients [35], and a recent cluster randomised trial showed that training palliative care nurses in CBT reduced patients' anxiety, but no significant effect was found for depression [37]. For problem-solving therapy studies are scarcer still, though its simplicity and brevity make it a popular choice in palliative care - as reflected in the experts' ratings. The item "Presence of spiritual assistant upon request" was suggested by an expert in the first round of Delphi, and then attained the third highest rating in the second round. Life-threatening illness poses existential challenges that can exacerbate distress at the end of life and surveys have indicated that around one third of cancer patients have unmet spiritual needs [38,39]. The expert group's endorsement of spiritual assistance as a useful intervention in palliative care is corroborated by a recent survey showing an association between spiritual care and better quality life near death [40]. However, the efficacy of spiritual care in specifically improving depression outcomes has not been determined, and nor is it likely that such an approach will be testable in a conventional RCT. Rather than see spiritual care as competing with conventional psychotherapies, it might be better conceptualised as an adjunct for those patients who wish to have it. Mindfulness-based therapy, guided imagery and narrative therapy were rated as moderately useful by the expert group (median 7) despite a lack of evidence in palliative care. Randomised controlled trials of these therapies are needed to provide an evidence base to support expert opinion.

### Strengths and limitations

Although modest, our 62% response rate is similar to that achieved in previous studies in which the Delphi method has been applied in palliative care research [17,41]. Several reminder emails were sent to the expert group as a whole, but we felt that pursuing non-responders individually would detract from the anonymity of the Delphi process.

The multinational nature of the expert group enabled the Delphi study to capture the views of experts from ten European countries. However, development of the guideline was led by a UK research group and there was a British bias in the composition of the expert group. As a consequence our findings may be more representative of the views of UK health care professionals, and less applicable to other European countries. The expert group was multidisciplinary to encompass the perspectives of the different professions involved in the management of depression in palliative care. Despite this, we acknowledge that certain disciplines, such as psychology and nursing may have been under-represented [42].

Previous studies have demonstrated the potential for the Delphi process to increase expert consensus (indicated by a reduction in the range of ratings from round to round). In this study the spread of expert responses tended to remain broad in both rounds, indicating entrenched differences in palliative care providers' views on how best to detect and treat depression. However, experts' ratings of the appropriateness of different antidepressants in palliative care indicated some convergence in opinion between round one and two. In round two there was an even sharper distinction between those antidepressants deemed appropriate in palliative care and those not (see Table 3). With the exception of escitalopram, the favoured antidepressants achieved an equal or higher median rating in round two compared to round one, whereas the antidepressants considered less appropriate, achieved an equal or lower median rating in the second round. Thus, the Delphi process contributed to a clearer picture of experts' opinion on choice of antidepressant. In relation to choice of screening tool and choice of psychological therapy, there was less evidence of convergence in expert opinion between round one and two. However our aim was to evaluate experts' views and assess the level of expert agreement, not to achieve consensus on clinical practice. The range of ratings in this study reveals wide variation in expert opinion, which is underscored by the contrasting comments clinicians left to annotate their scores. Our sample of experts was not sufficiently large to enable investigation of systematic differences in opinion based on characteristics such as clinical specialty, country or age. This could be a fruitful avenue for future research.

## Conclusions

The Delphi method is an efficient and effective way of eliciting expert opinion and promoting international collaboration. Experts with knowledge and experience of managing depression in palliative care were unconvinced about the benefits of screening and prefer to 'ask' patients about mood as part of routine symptom assessment. Mirtazapine and citalopram were the considered the best choice of antidepressant and cognitive behavioural therapy the best choice of psychological therapy. The range of expert ratings was broad, indicating discordance in the views of experts. There was little evidence of convergence of opinion in round two. The EPCRC depression guideline aims to prevent inappropriate variation in patient care by providing best practice recommendations on managing depression in palliative care. However, a paucity of high quality evidence precludes definitive statements regarding the optimal screening tool, antidepressant and psychological therapy. Direct comparative data are needed to strengthen the evidence-base and achieve clarity on how best to detect and treat depression in this setting.

## Competing interests

MH is an independent expert witness (instructed by the claimants' solicitor) in a group litigation on the potential for paroxetine to cause adverse events on withdrawal of treatment. LR, AP and IHJJ do not have any competing interests. No competing interests were declared by the members of the expert group.

## Authors' contributions

LR coordinated the study, including design of Delphi questionnaire, correspondence with experts, collation, analysis and interpretation of data and writing of the manuscript. AP participated in the design of the study and Delphi questionnaires. MH won peer review funding for project and supervised the study - including design of Delphi questionnaire, collation, analysis and interpretation of data, and writing of the manuscript. IJH won peer review funding for project and supervised the study - including design of questionnaire, correspondence with experts, collation, analysis and interpretation of data, and writing of the manuscript. All authors have read and approved the final manuscript.

## Pre-publication history

The pre-publication history for this paper can be accessed here:

http://www.biomedcentral.com/1472-684X/10/10/prepub

## Supplementary Material

Additional file 1 **Delphi questionnaire**. The questionnaire sent to the Expert Group for the first round of the Delphi process. Click here for file
