# Supplementary material for: Expert opinion on detecting and treating depression in palliative care: A Delphi study
Source: BMC Palliat Care. 2011 May 27;10:10. doi: 10.1186/1472-684X-10-10 (PMC3125275; doi:10.1186/1472-684X-10-10)
Supplement: Additional file 1 — Delphi questionnaire. The questionnaire sent to the Expert Group for the first round of the Delphi process. [file 1472-684X-10-10-S1.DOC]

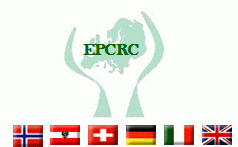

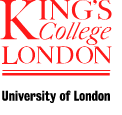


**WP 3.2: EPCRC Depression Guideline**

**Delphi Questionnaire: First Round**

Thank you again for your comments on the first draft of the EPCRC Depression Guideline.

Feedback from the Expert Group has revealed some areas of uncertainty within the scope of the Guideline. The issues which provoked debate have been incorporated in the following Delphi questionnaire. The purpose of this questionnaire is to assess the level of expert agreement with contentious issues relating to the detection and treatment of depression in palliative care in order to inform the EPCRC guideline. We kindly ask you to score each recommendation on a scale from 0 to 10, leaving additional comments in the space provided. Focus on those which address your area of expertise, and leave blank any that you do not feel able to answer. Delphi is an iterative process, involving repeated expert input and anonymous feedback of responses. Responses to the questionnaire will be analysed and fed back to the Expert Group. You will then be asked to consider whether you wish to keep or change your scores, in light of the results of the first round.

We would be grateful if you could complete and return the questionnaire by **Friday 26^th^ June.**

Thank you very much.

| 1: Choice of screening tool | **Usefulness**  **0 = not useful, 10 = very useful**  **Please, mark (X) one answer only** | | | | | | | | | | | **Comments** |
| --- | --- | --- | --- | --- | --- | --- | --- | --- | --- | --- | --- | --- |
|  | **0** | **1** | **2** | **3** | **4** | **5** | **6** | **7** | **8** | **9** | **10** |  |
| Single item question: “Are you depressed?” |  |  |  |  |  |  |  |  |  |  |  |  |
| Two-item question: “During the last month have you often been bothered by 1) feeling down, depressed or hopeless, 2) having little interest or pleasure in doing things?” |  |  |  |  |  |  |  |  |  |  |  |  |
| Hospital Anxiety and Depression Scale (HADS) |  |  |  |  |  |  |  |  |  |  |  |  |
| Primary Care Evaluation of Mental Disorders (PHQ9) |  |  |  |  |  |  |  |  |  |  |  |  |
| Beck’s Depression Inventory (BDI) |  |  |  |  |  |  |  |  |  |  |  |  |
| Brief Edinburgh Depression Scale (BEDS) |  |  |  |  |  |  |  |  |  |  |  |  |
| Distress Thermometer (DT) |  |  |  |  |  |  |  |  |  |  |  |  |
| Question(s) on depression as part of a generic symptom assessment scale (e.g. the Palliative care Outcome Scale (POS), the Edmonton Symptom Assessment Scale (ESAS) or an overall quality of life scale (e.g. EORTC QLQ) |  |  |  |  |  |  |  |  |  |  |  |  |
| Other (please list) |  |  |  |  |  |  |  |  |  |  |  |  |
|  | | | | | | | | | | | | |

| 2. Choice of psychological therapy | **Usefulness**  **0 = not useful, 10 = very useful**  **Please, mark (X) one answer only** | | | | | | | | | | | **Comments** |
| --- | --- | --- | --- | --- | --- | --- | --- | --- | --- | --- | --- | --- |
|  | **0** | **1** | **2** | **3** | **4** | **5** | **6** | **7** | **8** | **9** | **10** |  |
| Cognitive Behavioural Therapy (CBT) |  |  |  |  |  |  |  |  |  |  |  |  |
| Problem-solving Therapy |  |  |  |  |  |  |  |  |  |  |  |  |
| Guided imagery |  |  |  |  |  |  |  |  |  |  |  |  |
| Couple Therapy |  |  |  |  |  |  |  |  |  |  |  |  |
| Interpersonal Therapy |  |  |  |  |  |  |  |  |  |  |  |  |
| Mindfulness-based therapy |  |  |  |  |  |  |  |  |  |  |  |  |
| Narrative Therapy |  |  |  |  |  |  |  |  |  |  |  |  |
| Other (please list) |  |  |  |  |  |  |  |  |  |  |  |  |
|  | | | | | | | | | | | | |

| 3. Choice of antidepressant | **Usefulness**  **0 = not useful, 10 = very useful**  **Please, mark (X) one answer only** | | | | | | | | | | | **Comments** |
| --- | --- | --- | --- | --- | --- | --- | --- | --- | --- | --- | --- | --- |
| **Selective Serotonin Reuptake Inhibitors**  **(SSRIs)** | **0** | **1** | **2** | **3** | **4** | **5** | **6** | **7** | **8** | **9** | **10** |  |
| Sertraline |  |  |  |  |  |  |  |  |  |  |  |  |
| Citalopram |  |  |  |  |  |  |  |  |  |  |  |  |
| Fluoxetine |  |  |  |  |  |  |  |  |  |  |  |  |
| Escitalopram |  |  |  |  |  |  |  |  |  |  |  |  |
| Paroxetine |  |  |  |  |  |  |  |  |  |  |  |  |
| **Tricyclics** |  |  |  |  |  |  |  |  |  |  |  |  |
| Amitriptyline |  |  |  |  |  |  |  |  |  |  |  |  |
| Nortriptyline |  |  |  |  |  |  |  |  |  |  |  |  |
| Impipramine |  |  |  |  |  |  |  |  |  |  |  |  |
| **Other types of antidepressant** |  |  |  |  |  |  |  |  |  |  |  |  |
| Mianserin |  |  |  |  |  |  |  |  |  |  |  |  |
| Mirtazapine |  |  |  |  |  |  |  |  |  |  |  |  |
| Venlafaxine |  |  |  |  |  |  |  |  |  |  |  |  |
| **Other (please list)** |  |  |  |  |  |  |  |  |  |  |  |  |
|  | | | | | | | | | | | | |

| **Please make any general comments here** |
| --- |
|  |

**Please email your completed questionnaire to** [**Lauren.Rayner@kcl.ac.uk**](mailto:Lauren.Rayner@kcl.ac.uk)

**Thanks again for your support.**
